# Uterus Transplants and the Potential for Harm: Lessons From Commercial Surrogacy

**DOI:** 10.1111/dewb.12274

**Published:** 2020-07-06

**Authors:** Gulzaar Barn

**Keywords:** Uterus transplant, commercial surrogacy, consent, exploitation, structural justice

## Abstract

The human uterine transplant (UTx) is being developed as a procedure to alleviate absolute uterine factor infertility (AUFI). In light of recent UTx advances in India, I suggest that key ethical concerns would emerge if this procedure were to become established as part of India’s liberalised assisted reproduction industry. On evidence-based projections, UTx would be likely to harm vulnerable populations in two key ways. Firstly, I suggest that a commercial model for uteri procurement is primed to develop due to various structural factors that facilitated the boom in commercial surrogacy remaining in place, despite commercial surrogacy having been outlawed. I outline ways in which Indian commercial surrogacy arrangements exhibited exploitation and suggest that a commercial UTx model would be similarly exploitative, with many background features of exploitative surrogacy remaining constant. The second way in which the development of UTx in India might pose harm is through the difficulty in obtaining proper informed consent, even under altruistic arrangements. I argue that structural factors, including a cultural deference to doctors, lack of medical understanding displayed by vulnerable populations, and a privatised healthcare system with little regulatory oversight, would render it difficult to obtain proper informed consent from living donors. Further, factors peculiar to UTx, such as its experimental nature, and the unknown and novel risks and harms it poses, heighten this difficulty. Accordingly, UTx in India would be unlikely to meet the Montreal criteria for the ethical feasibility of uterine transplantation and should thus raise ethical alarm.

## 1 Introduction

There have been significant recent advances in the development of the human uterine transplant (UTx) as a procedure to alleviate absolute uterine factor infertility (AUFI). Since the first UTx study in Sweden in 2012, fifty-six transplants have taken place worldwide, leading to fourteen live births.^1^ It has been suggested that the most significant obstacles to the availability of UTx are presently scientific and technical, relating to the safety and efficacy of the procedure itself.^2^ If and when such obstacles are overcome, the most likely barriers to its availability will be social and financial in nature, relating to how funding for such procedures would be provided.^3^ A further concern relates to how to source enough uteri for research and transplant purposes, and whether living or dead donors should be preferred.

Given recent global trends in assisted reproductive technologies and transplant tourism, UTx and uterine donation may also grow to be regulated by market norms. This seems more likely to occur in less economically developed countries with high poverty rates, creating a willing pool of vendors, and liberalised private health care systems, factors that have contributed to the development of commercial surrogacy in countries such as India. In this paper I advance the idea that if UTx were to develop in India – and the first transplants have already been performed there^4^ – key ethical concerns would be likely to emerge, in the form of harms to vulnerable populations. Firstly, I suggest that a commercial model for uteri procurement is primed to develop, due to various structural factors that facilitated the boom in commercial surrogacy remaining in place, despite commercial surrogacy having been outlawed. Indian commercial surrogacy arrangements have been criticised for being exploitative, and I suggest that a commercial UTx model would be similarly likely to exhibit features of exploitation.^5^ The second way in which the development of UTx in India might pose significant harm is through the difficulty in obtaining proper informed consent in this context. Setting aside the possibility of commercialisation, I suggest that structural factors, such as a cultural deference to doctors, lack of medical understanding displayed by vulnerable populations, and privatised healthcare system with little regulatory oversight, render it difficult to obtain proper informed consent from living donors. I draw upon research suggesting that rural women have been subject to medically unnecessary hysterectomies^6^ in order to shed light on the predatory nature of the healthcare system in India, and the way in which vulnerable populations are not suitably safeguarded in terms of consent and due care. This background context may similarly motivate harmful practices emerging relating to UTx. Further, factors peculiar to UTx, such as its experimental nature, as well as the novel risks and harms it poses, also contribute to the difficulty in obtaining proper informed consent in this context. As a result, UTx in India would not meet the Montreal criteria for the ethical feasibility of uterine transplantation and should thus raise ethical alarm.^7^

The Montreal criteria are a set of “proposed criteria required for a woman to be ethically considered a candidate for uterine transplantation.”^8^ They are developed in the context of considerations relating to experimentation and standard practice and emphasise the particular importance of obtaining consent in clinical research, a category that UTx falls into. The criteria stipulate that the donor must be “responsible enough to consent, informed enough to make a responsible decision, and not under coercion.”^9^ I argue that donor consent to UTx in India would not satisfy these criteria.

The argument concerning uterine sales and exploitation is, as it stands, hypothetical, as no such markets presently exist. However, the aim of drawing the parallel with commercial surrogacy is to forewarn about the possibility of, and thus, prevent, similar exploitation concerns emerging with UTx, before it unfolds on a larger scale. In doing so, I seek to emphasise the importance of context-sensitive ethical inquiry. The argument relating to the difficulty in obtaining proper informed consent for UTx, however, pertains to the existing altruistic donation model for UTx in India.

## 2 The Advancement Of Utx

I begin by sketching out why it would be plausible to hypothesise that uteri could be procured according to a commercial model in India. We are witnessing global advancements in UTx: the first baby born from UTx using a dead donor was born in Brazil in December 2018,^10^ and a number of successful births using living donors have occurred since the first in Sweden in 2014.^11^ The use of dead donors could greatly broaden access to UTx, in terms of increasing the pool of uteri available, as live donors are rare and typically tend to be family or close friends. Some states permit only the use of uteri from deceased donors, while countries such as Sweden prefer living donations.^12^ Living donors are sometimes preferred for medical reasons relating to transplant success, although there remains disagreement about whether donations from living or dead donors have a higher chance of success.^13^ The use of living donors raises novel concerns, as this approach is “both necessarily harmful to donors *and* risks the possibility of failures to attain fully informed consent and donor regret.”^14^

One way that states might seek to increase the number of uteri available for transplantation is by permitting their sale. As such procedures become increasingly successful, increased donations may be required to enable UTx to become an established treatment for uterine factor infertility. Payment could serve as an incentive for donation. Janet Radcliffe Richards has made such an argument regarding kidney sales, suggesting that our current approach of total prohibition should be questioned in the context of widespread kidney shortages.^15^ John Harris and Charles A Erin make a similar argument regarding the use of sales to increase availability: “while people’s lives continue to be put at risk by the dearth of organs available for transplantation, we must give urgent consideration to any option that may make up the shortfall. A market in organs from living donors is one such option.”^16^

While such arguments for markets in kidneys have not been put into practice (the only country which legally permits kidney sales is Iran^17^), I introduce them to show that payment is sometimes suggested in the literature as a means of increasing the supply of organs for transplantation, and that similar arguments may be made in the case of UTx. Further, because a uterus may not be seen as necessary for one’s overall health, it might seem less onerous for vendors to part with it, thus increasingly the acceptability of such sales. Later, however, I scrutinise this latent conception of the ‘expendable’ nature of the uterus, suggesting that the permanent loss of an organ and its attendant functioning poses unique problems for obtaining consent to such living donation. I suggest that this problem holds for unpaid voluntary uteri donations too, which are presently legal.

Sweden is pioneering UTx research, using living donors. Surrogacy is not permitted in Sweden, either on an altruistic or a commercial basis, but children have been born to Swedish citizens using cross-border surrogacy.^18^ In the Swedish context, UTx is sometimes heralded as an approach that can enable childless couples to have genetically-related children, without the need to involve a gestational surrogate, and thus appears to be regarded as less ethically fraught^19^ – though, as I will argue, this supposition is questionable. Given the highly restrictive nature of practices such as surrogacy and gamete sales in Sweden, it is unlikely that a commercial model for uterine donation would develop there. However, a Swedish research team successfully worked with doctors in Lebanon to perform Lebanon’s first UTx transplant in July 2018.^20^ Of note, since surrogacy is prohibited in Lebanon,^21^ UTx could be in high demand there as a route towards genetic parenthood. A commercial model currently exists for gamete distribution within Lebanon,^22^ and the country’s healthcare system is dominated by the private sector.^23^ Furthermore, there have been recent reports that Syrian refugees living in Lebanon have resorted to selling their kidneys and in some cases, eyes, in an illegal organ trade.^24^ Many refugees are not permitted to work under Lebanese law, and there is limited access to aid and other supplies. Since 2011, Lebanon has taken in 1.5 million Syrians refugees and has the highest concentration per capita of refugees in the world.^25^ Given the existence of trades in other organs in Lebanon, the development of an underground market in uteri seems possible.

However, India may be most primed for the development of uterine sales. India has become a world leader in surrogacy and assisted reproduction services. The first baby to be born through UTx in India was successfully delivered in October 2018^26^ and a recent commentator suggests that UTx is poised to become part of India’s booming assisted reproductive technology (ART) industry.^27^ Sandhya Srinivasan argues that UTx is being presented in India as an established practice despite its risky and highly experimental nature, and that there are significant gaps in its regulation.

Srinivasan notes that there is no body in India that is formally charged with the regulation of UTx as research.^28^ If UTx were an established therapeutic procedure, it would be regulated under the Transplantation of Human Organs and Tissues Rules Act (THOTA). In the case of UTx, by convention, the Indian Council of Medical Research (ICMR) would be responsible for developing ethical guidelines on UTx research, and such research would be required to be reviewed and monitored by the institution’s ethics committee. However, neither the ethics committee nor the ICMR has the authority to take any substantial action against violations in such trials; nor is compensation required for participants who are injured or die. The surgical team in the first UTx in India in 2017 did not include a surgeon with the THOTA-specified “one year training in the respective organ transplantation as an active member of team in an established transplant center”^29^ – among the requirements for certification as a transplant hospital. The ICMR’s director general has admitted that uterus transplantation is a “grey regulatory area.”^30^

Referring to the surrogacy industry and the sale of ova, Srinivasan observes that “the ART industry in India has a well-documented history of unethical practice and a healthy dislike of regulation.”^31^ Further, such an industry thrives in a sociocultural context that places great pressure on women to have genetically-related children – a situation that remains in place for, and indeed, plausibly motivates, the development of UTx. Srinivasan warns that this pressurised environment makes it “unlikely that women will be able to make a truly informed decision on whether or not to risk their health” and that it seems “more than possible that family members can be coerced into donating their uterus, possibly even for payment.”^32^ I return later to explore the possibility of exploitation and the difficulty of obtaining informed consent in this context.

The lack of clarity regarding regulation that allowed surrogacy to develop and thrive according to a highly commercialised model, under which ART clinics were able to operate according to their own rules, could lead to a similar situation unfolding with UTx. Furthermore, the sociocultural demands for genetic parenthood remain in place. If it were to develop, a commercial model is likely to exhibit features of exploitation, in the same way that surrogacy in India typically has done.^33^ I will now explain how various features of the Indian surrogacy context contributed to its exploitative nature and outline how UTx may follow suit.

It may be objected at this point that a commercial model is simply unlikely to develop for UTx. Of note, India has banned surrogacy on a commercial basis, realising its potential for exploitation. Analogously, organ sales are not legally permitted in India; therefore it would seem that a similarly restrictive approach would extend to the regulation of uteri. However, a ban is arguably ineffective in a context that permits various incidences of illegality and medical wrongdoing. As I will argue, the widespread use of financially motivated medically unnecessary hysterectomies suggests that doctors in the Indian context are willing and able to act in ways that contravene good medical practice and the law. Moreover, although India has changed its laws surrounding surrogacy, reports have emerged that clinics flout restrictions by moving Indian surrogates across borders, to countries such as Nepal, and by recruiting Kenyan surrogates into Indian clinics and flying them to Kenya to give birth.^34^ Given that altruistic surrogacy will still be permitted, and ART clinics will still be in operation, it seems plausible to hypothesise that similar such loopholes will be sought, and that surrogacy could continue to operate as a commercial enterprise, below the radar. This would suggest that a black market in uteri procurement is similarly possible. To illustrate further, although organ sales are not permitted in India, according to the World Health Organisation (WHO), South Asia is the leading transplant tourism hub globally, with India among the top kidney exporters.^35^ Each year more than 2,000 Indians sell their kidneys. It is reported that potential donors are sent for pre-arranged blood tests and a tissue-typing test in chosen pathology laboratories across India, or sent to large private speciality hospitals, where doctors are aware of, and indeed, part of the illegal kidney networks.^36^ Doctors and clinics, it seems, are willing and able to undermine legal guidelines where they exist. We should be mindful of similar motivating factors, regarding supply and demand, existing in relation to uterine procurement.

## 3 The Indian Context: The Shadow of Commercial Surrogacy

### 3.1 Background

In this section I provide some background to the Assisted Reproductive Technology (ART) in industry in India. Following this, I offer a definition of exploitation and present evidence suggesting that Indian commercial surrogacy arrangements typically satisfy the exploitation conditions specified. Thereafter, I suggest that the background conditions comprising Indian surrogacy arrangements remain in place, and would thus result in similarly exploitative transactions, if a commercial model for UTx were to emerge.

India emerged as a leader of commercial surrogacy arrangements partly as a result of the relatively low-cost nature of its ART services. Until it was outlawed in December 2018,^37^ the total cost of surrogacy in India could be around £18,000, of which the surrogate typically receives around £4,000.^38^ There was no legally enshrined minimum wage for surrogacy, so the fees paid to surrogates varied. Surrogacy in India is about a third of the price of surrogacy in America, which costs around $80,000 (£60,000), with American surrogates typically receiving $35-40,000 (£26-30,000).^39^ Commercial surrogacy is not legal in the UK, but surrogacy is permitted on an altruistic basis, where surrogates are reimbursed reasonable costs.^40^ In December 2018, India’s lower House of Parliament passed a new version of the Assisted Reproductive Techniques Regulation Bill which bans all surrogacy on a commercial basis, permitting only altruistic surrogacy to Indian couples on a case-by-case basis.^41^

In order to understand how a transnational commercial surrogacy industry came to flourish, and how a commercialised UTx model might similarly develop, it is necessary to locate surrogacy in India’s wider medical tourism agenda. Dominated by the private sector, India’s health care industry has been growing at an annual rate of almost 15%.^42^ Spending 4.7% of GDP on healthcare, public investment into health care in India is among the lowest in the world.^43^ In order to attract foreign capital, the Indian government actively promotes medical tourism, with assisted reproductive services being a key aspect of this. It does this through providing financial incentives to private hospitals, reducing import tariffs for medical equipment, and expediting medical visas and joint insurance collaborations in order to facilitate medical travel.^44^ Commercial surrogacy was legalised in 2002, but, until recently, was regulated only by non-binding guidelines, issued by the Indian Council of Medical Research (ICMR) in 2005. It is important to remember that this liberalised healthcare context remains in place for Indian trials of UTx. Later, I present evidence suggesting that private hospitals have been operating according to financial incentives by providing medically unnecessary hysterectomies to vulnerable Indian women, and that such procedures have failed to meet standards of informed consent. This lends support to the charge that informed consent conditions would not be met if UTx became an established procedure.

### 3.2 Exploitation

The language and content of India’s new surrogacy Bill suggests that addressing the exploitation of surrogates was a key motivating factor in its development. Indeed, Part VII of the Bill specifically outlines the “prohibition of commercial surrogacy, exploitation of surrogate mothers and children born through surrogacy,”^45^ and the term ‘exploitation’ features several times in the Bill. In the following section, I set out various features of the Indian situation, which I take to be morally salient, and critical to the charge of exploitation. It does not undermine the argument I intend to make – relating to the potential for exploitative UTx practices to develop – that India has declared a ban on international surrogacy, seemingly to address the issue of exploitation. Indeed, India’s decision may even serve to support my argument that the empirical reality suggests Indian surrogacy arrangements *were* typically exploitative. Importantly, I show that the background conditions that comprised such exploitative arrangements remain in place. The prohibition of commercial surrogacy does not ensure that UTx would not develop according to a commercial model. Rather, I suggest that current regulatory loopholes may encourage the development of UTx now that surrogacy is no longer a legal option.

A preliminary step to invoking exploitation in relation to the case of commercial surrogacy is to develop an analysis of exploitation, determining the conditions under which exploitation can be said to occur. Following this, the task will be to establish whether commercial surrogacy arrangements satisfy these criteria. There is limited scope to develop such an account in this paper – exploitation is of course an “essentially contested”^46^ concept, in that there are genuine, irresolvable disputes about its proper use. However, I tentatively deploy the definition *A exploits B when A takes unfair advantage of B’s vulnerability*. There seems to be general consensus within the literature that exploitation tends to have two components, relating to process and outcome. Exploitation necessarily involves some unfairness. This unfairness is said to be either procedural or substantive. Procedural unfairness is the result of some procedural defect in the transaction: “*A* unfairly utilises or creates a defect in the process of the transaction with *B* in a way that benefits *A* at *B*’s expense.”^47^ So, when A takes unfair advantage of B’s *vulnerability*, this is an instance of procedural unfairness. Without the procedural defect of *taking advantage of vulnerability*, the transaction might not have been unfair, or indeed, it may not have been agreed to by B at all, if B had not been vulnerable. The vulnerability element of my definition, therefore, refers to the *process* by which the transaction occurred, and the unfairness contained therein. To illustrate, when an elder brother “trades five pennies for his 5 year-old brother’s single quarter,”^48^ he unfairly takes advantage of his younger brother’s vulnerability, consisting in his ignorance of monetary units, thus generating a procedural defect.

With substantive fairness, “the unfairness is a feature of *what* is agreed to, rather than *how* the agreement is reached.”^49^ Broadly speaking, therefore, the substantive unfairness pertains to the issue of unfair price, or an unequal exchange. On such a view, a transaction might be unfair because A benefits disproportionately to B, taking a much higher proportion of the transaction’s social surplus. This is reflected in the “unfair advantage” component of my definition.

I conceive of ‘vulnerability’ broadly and with reference to a minimal justice requirement,^50^ the non-satisfaction of which will render B vulnerable in the sense relevant to this conception of exploitation. One is vulnerable, therefore, in accordance with a conception of justice relating to the basic structure of society. That is, vulnerability necessarily results from failures in the justice of the basic structure. Vulnerability represents various degrees of disadvantage, across different categories. Such categories may include educational status, income, health, disability, gender, and race. These categories can intersect to create variable experiences of disadvantage. One can be vulnerable in various ways, to various degrees, and there is a sense in which human beings, as a result of their inherently deteriorative embodiment, are universally vulnerable. Despite this universality, it remains possible to classify some individuals as *more* vulnerable, compared with others. It is desirable to retain vulnerability’s status as a *scalar* notion, while acknowledging its universality, in order to reasonably conclude that by and large, and according to numerous measures, surrogates in India are particularly vulnerable.

I accept that such a definition may be susceptible to counterexamples and may not be able to accommodate every case of exploitation. The contested nature of the debate is such that any account may end up being too under-or over-inclusive. This account is intended to be generally acceptable, not universally so; therefore I acknowledge that difficult cases and questions will remain. Nevertheless, the definition I employ is appropriate to the purposes of this analysis: it serves as a useful philosophical tool for exploring what is objectionable about Indian commercial surrogacy arrangements, and analogous cases, such as uterine and other organ sales. I will now outline the ways in which the Indian situation satisfies the above definition of exploitation. I will argue that surrogates’ backgrounds generate the procedural defect that makes them vulnerable to exploitation, and the unfair advantage element of the definition is satisfied by the unfair terms of contract, including low payment and the risk posed by the types of procedures used.

Importantly, this exploitation is to be considered *harmful*, as opposed to *mutually advantageous*, on the dichotomy put forward by Alan Wertheimer.^51^ For Wertheimer, this distinction between mutually advantageous and harmful exploitation gives rise to a verdict regarding whether a particular case of exploitation is permissible or not. A practice being a case of mutually advantageous exploitation is unlikely to establish that it should be prohibited. Rather, the case for intervention is only raised by harmful exploitation, where the exploitee suffers a *net harm*. I suggest that surrogacy in India is more likely to be typically *ex ante* net harmful to surrogates, due to the lack of informed consent at various stages of the process, the nature of various medical practices used, which go against accepted protocol, as well as the use of surrogacy hostels. These factors outweigh the benefit gained by surrogates, rendering commercial surrogacy arrangements in India typically harmful. I then suggest that these systemic factors remain in place and could plausibly fuel the development of deregulated UTx services, that would also satisfy this procedural and outcome-based exploitation condition.

#### 3.2.1 The vulnerability component

The Indian surrogacy industry is characterised by wealthy international clients procuring the services of Indian surrogates who are comparatively and absolutely less well off, their socioeconomic status comprising their vulnerability. Amrita Pande, one of the leading ethnographers in this area, has conducted extensive fieldwork in Anand, Gujarat, a city considered the global surrogacy capital. She found that 34 of the 42 surrogates that she interviewed reported family income below or around the poverty line,^52^ and “all but one reported acute financial desperation.”^53^ Pande also found that for most of the surrogate’s families, the money earned through surrogacy was “equivalent to almost five years of total family income.”^54^ Such statistics illustrate the profound poverty of the women who are choosing to become surrogates, and their palpable financial motivations. Pande notes that of the women she interviewed, most “were driven to surrogacy because of financial desperation, often compounded by a medical emergency and an urgent need for liquid cash.”^55^ In her six years of fieldwork, she only came across two women who identified as middle class. Pande’s other findings about the women’s backgrounds revealed that fourteen were “housewives,” two “worked at home,” and the others worked at schools, clinics, farms and stores. Their education ranged from illiterate to high school level, with the average being middle school level.^56^ Many of their husbands were either in informal work such as rickshaw pullers, factory workers, and stall workers, or unemployed. Research by Daisy Deomampo, another ethnographer working in the area of Indian commercial surrogacy, appears to corroborate Pande’s findings: “in general the surrogates and egg donors who participated in my study had low rates of access to education; many stopped school by seventh grade.”^57^

Interviewing surrogates from four different surrogacy clinics, Tulsi Patel and Sunita Reddy also found that surrogates’ “primary motives were the meager earnings of their husbands, debts, and family responsibilities. Other reasons included domestic violence and alcoholism.”^58^ A doctor interviewed also “stated that the main reason surrogates come forward is because of financial hardship.”^59^ Corroborating this, a surrogacy agent said the following about surrogates: “they are really needy, poor and helpless. Many women want to commit suicide because of financial burdens. In the last 3 years at least three to four hundred women have approached me.”^60^ Surrogates in India might be considered vulnerable, and thus satisfy the vulnerability component in standard definitions of exploitation, due to factors such as poverty, illiteracy, and a lack of other earning options.

Surrogates’ vulnerability can also be seen in their position in a social class that has historically been subject to fertility control. Sharmila Rudrappa, who has also conducted extensive fieldwork with surrogates in India, notes that India’s history of *fertility control* and *fertility assistance* are connected. Rudrappa locates surrogacy in the history of medical interventions in order to “reveal the long-term patterns of control over working-class women’s bodies.”^61^ Most of the surrogates Rudrappa interviewed had themselves been sterilised. Similarly, Pande notes that of the surrogates she interviewed, 13 of the 20 that practised some form of contraception were in fact sterilised.^62^ This fits into a wider trend: 37% of married women in India opted for tubal litigation between 2005-6, and sterilisation is the most prevalent form of “contraception” among rural women and those with lower levels of education.^63^ The prevalence of sterilisation as a form of birth control is located in India’s history of population control programmes.^64^ After its independence from Britain in 1947, concerns about population size and a desire to modernise led India to implement what would become the largest state-sponsored family planning programme in the world.^65^ Methods of birth control such as intrauterine devices (IUDs) and long-acting hormone-based contraception such as anti-fertility vaccines were aggressively propagated^66^ as long-term and more invasive contraceptive methods were pushed as the requirements of the pill regimen were seen to be “beyond the powers of mind and discipline of illiterate Indian housewives.”^67,68^ Coerced sterilisation underlines the disadvantage faced by this section of the population, and is relevant to the assessment of surrogacy because it further supports the idea that surrogates are vulnerable, a status that is key to their exploitation. It is no coincidence that many of the women who are choosing to become surrogates have also been sterilised, plausibly as part of India’s class-based population control programmes: they occupy a historically targeted position in society.

There is reason to believe that a market in uterine sales, either underground or legalised, would also be exploitative. This is because it would be likely to exhibit similar features relating to *process* – i.e. the vulnerability of the sellers, and *outcome* – the unfair advantage gained by the clinics in relation to the sellers. Regarding process, a similar pool of sellers could be available for uterine sales, as there was for surrogacy. Given the evidence on the backgrounds and motivations of those who choose to sell a kidney^69^ or become a surrogate, it seems plausible that those most disposed to sell a uterus would also be vulnerable, in regards to poverty, lack of education, and lack of other suitable earning options, conditions that I have identified as key to exploitation in surrogacy arrangements.

Further, as aforementioned, Indian surrogacy developed in a context that restricted the reproductive autonomy of the poor – surrogates belong to a class that has historically been subject to fertility control. Such a pool has already been targeted through sterilisation; accordingly, individuals from this cross-section of society might well divest of their uterus for UTx, either through persuasion, payment, or the kind of coercion highlighted above. If sterilisation is routinely encouraged as a means of birth control by the Indian medical profession, further medically unnecessarily or non-standard practices related to uterine procurement could develop, given a context that appears to normalise access to vulnerable women’s bodies in this way. This issue will be explored further in the Informed Consent section of this paper, where I highlight the potential for UTx-related harmful practices to develop, in light of the widespread use of medically unnecessary hysterectomies amongst impoverished rural women, and controversial ART practices in the surrogacy context.

#### 3.2.2 The ‘unfair advantage’ component

The Indian surrogacy industry seems to fulfil the *outcome* element of exploitation, related to the ‘unfair advantage’ gained over surrogates, in the following way. Due to a lack of regulation, Indian surrogacy developed in a context where clinics were able to operate by their own rules, resulting in contracts that prioritise the ease of the transaction, rather than enshrining protection for surrogates. The absence of legal provisions means that the surrogate is not afforded protection in the event that the pregnancy affects her own health. Further, if the pregnancy results in a miscarriage or deformity of the infant, there is nothing in the law to ensure the surrogate receives the promised amount at the end of the surrogacy.^70^

A key outcome-related issue that renders the arrangements exploitative is the *nature* of some of the procedures used. In India the medical practices used raise particular ethical concern. Writing generally, Alan Wertheimer notes that there are certain objective harms associated with surrogacy: “the risk of physical harm or death resulting from the pregnancy or the delivery, restraints on the surrogate’s choices during pregnancy, and the inconvenience and discomfort associated with a normal pregnancy,”^71^ but that these ought to be considered on a case by case basis alongside the benefits, to decide on balance, whether surrogacy is net harmful or beneficial. Restraints on choices and discomfort both appear to be inherent to pregnancy and thus are harms likely to be experienced by almost all surrogates. The Indian context features additional risks and harms, however. As the focus is on delivering the best service to customers, the surrogates themselves appear to be deprioritised. This can be seen in the use of various controversial medical practices.

In the UK, the National Institute for Health and Care Excellence (NICE) guidelines recommend single embryo transfer and state that doctors should only consider using two embryos if no top-quality embryos are available.^72^ Similarly, American Society for Reproductive Medicine guidelines state that doctors should implant just one, and certainly no more than two, embryos at a time.^73^ In India, the Assisted Reproductive Technologies Rules (2010) acknowledge that specific efforts must be made to reduce the incidence of multiple pregnancies but permit the transfer of three embryos at a time.^74^ The investigative journalist, Scott Carney, found that the Akanksha Infertility Clinic in Anand, Gujarat, routinely implants as many as five embryos at a time in a surrogate’s uterus,^75^ vastly exceeding both the Indian guidelines and the wider international consensus on what is safe. As a way of mitigating the risk of multiple pregnancies in cases where multiple embryos take, some are selectively aborted in the first trimester of gestation in a practice called multifetal reduction. This involves the insertion of a needle, guided by ultrasound, abdominally or vaginally, to inject potassium chloride into the foetus, stopping its heart.^76^ Carney found that in cases where three or more embryos take, the Akanksha clinic selectively aborts specific embryos to bring down the total to more manageable levels, often without explaining to surrogates what is happening.^77^

Further, caesarian sections are routinely used for ease of delivery and exerting control over its timing, despite carrying unique medical risks. Carney found that in a surrogacy hostel he visited in Anand, no surrogate he interviewed expected a vaginal birth.^78^ An investigative report by the San Francisco Chronicle, which also followed the Akanksha Infertility Clinic in Anand, found that three quarters of the 649 babies born there were delivered by caesarean.^79^ Pande found that although most women had their previous deliveries at home naturally, with minimal medical intervention, as surrogates they inevitably underwent a caesarean section: only two surrogates out of the 42 in her study had vaginal deliveries.^80^ Similarly, the ethnographer Sharmila Rudrappa found that almost all surrogates in her study “had delivered, or were scheduled to deliver babies, through caesarean surgeries between the thirty-sixth and thirty-eighth weeks of their pregnancies,”^81^ despite almost all of them having delivered vaginally for their own births. Home deliveries, of course, present their own risks, and their prominence amongst this group plausibly results from such women being economically barred from hospital healthcare. However, it remains that the routine use of C-sections and other contentious medical practices present serious tangible harms to surrogates, comprising the outcome-focused “unfair advantage” element of the exploitation.

The widespread use of such controversial medical practices is a result of various structural factors present in the Indian context, including the highly commercial, profit-driven nature of the ART industry, the recruitment of vulnerable populations, and a cultural deference that exists towards doctors and the medical profession. While commercial surrogacy may be outlawed, these structural factors remain in place, and are likely to generate analogous concerns regarding UTx and the deprioritisation of the donor. The terms of contract may similarly favour clinics, given the holes in the regulation of UTx that were outlined earlier. To recall, there is no body in India that is formally tasked with the regulation of UTx as research. As it is not yet an established therapeutic procedure, it is not formally regulated under the Transplantation of Human Organs and Tissue Rules Act (THOTA), which leaves clinics with leeway in terms of how they proceed, until this regulatory gap is closed. Indeed, the first UTx procedures were performed in an Indian hospital by a surgical team that did not include a surgeon with the THOTA-specified one year training in the respective organ transplantation as an active member of team in an established transplant centre.^82^ The Swedish doctor, Mats Brännström, who performed the world’s first successful uterus transplant surgery, warned that the womb transplant being attempted by surgeons at a this hospital was with “no proper preparations at all” and would put the patients at a “very high risk.”^83^

In conjunction with a Swedish team, another Indian clinic successfully sought permission from Indian Council for Medical Research (ICMR) for a temporary permit to conduct UTx procedures. However, Srinivasan suggests that the ICMR’s response and approval of this surgery violates key ethical and procedural requirements. The Health Ministry Screening Committee (HSMC) accepted the clinic’s institutional ethics committee approval, instead of sending the proposal its central ethics committee, which is specifically tasked with reviewing proposals forwarded to it by the ICMR. Further, the ICMR stated that the trial would be monitored by “the Ethics Committee and as per the guidelines of the DCGI (office of the Drugs Controller General of India),” though the DCGI has no authority for non-drug research. The ICMR has also refused to release a copy of the Bengaluru clinic’s application-related documents, including the approvals of the ethics committee and other regulatory bodies, information on previous research, expert opinions, and the informed consent form.^84^

The lack of a clear law regarding UTx raises procedural and ethical concerns. It was a lack of formal regulation that allowed clinics to follow their own guidelines in the development of commercial surrogacy, creating a model that was profit-driven and prioritised the interests of the clinics over those of the surrogates. Given the ART industry’s performance of non-standard medical practices in the context of surrogacy, it would seem that similar issues could arise with UTx as it develops in a context of regulatory gaps and opportunities for commercial enrichment. As aforementioned, Indian trials of UTx are already going ahead without sufficient ethical or regulatory oversight. Many of the conditions that led to an exploitative commercial surrogacy industry, therefore, remain in place for the development of a similarly concerning UTx industry.

## 4 Informed Consent

In this section I outline the ways in which UTx in India would be unlikely to meet standards of informed consent, and thus pose harm to donors. Factors which contribute to this inability to meet minimum consent requirements include a lack of medical knowledge and understanding displayed by vulnerable populations, a deregulated healthcare system, and the cultural deference displayed to medical professionals. This has led to practices developing in a way that prioritise commercial gain as opposed to safeguarding the interests of vulnerable populations. I draw a further parallel with the commercial surrogacy industry, which I suggest typically displayed defects in consent. Following this, I argue that uterus donation poses unique risks and harms, relating to permanent loss of functioning and gender identity, which may make consent harder to obtain, particularly in the Indian context, where I argue vulnerable populations are less able to control the amount of information they receive. These failures in consent are likely to arise in the context of both paid and altruistic uteri procurement, as they are peculiar to the nature of uterus donation in India, and are tethered to systemic factors independent of payment. Finally, I draw upon evidence revealing the routine use of medically unnecessary hysterectomies on rural Indian women, a situation that reveals a stark absence of informed consent. It has been suggested that the use of hysterectomies in this way is the result of “patriarchal bias, professional unscrupulousness and pro-private healthcare policies.”^85^ Importantly, these factors remain in place as UTx is trialled and in development – as a result, similar failures in consent would be likely to arise.

### 4.1 Surrogacy and informed consent

The lack of informed consent at various stages of the surrogacy process has been alluded to in the discussion so far. Failures in obtaining proper informed consent can be seen in the use of controversial medical practices, often performed without surrogates’ knowledge or understanding, as well as in various information failures surrounding the terms of the contract, with insufficient information having been disclosed to surrogates. In this section I suggest that proper informed consent will be similarly hard to obtain on the part of donors/potential vendors in the context of UTx, given the similarities outlined between the likely participants in each case, and the persisting structural factors. Moreover, factors peculiar to UTx, such as its experimental nature, as well as the novel risks and harms it poses, mean that proper informed consent might be even harder to obtain in this context. Following this, it appears that UTx in India would not meet the Montreal criteria for the ethical feasibility of uterine transplantation.^86^

As noted above, controversial medical practices such as the selective aborting of foetuses when multiple embryos take, have been performed without surrogates’ knowledge or understanding, constituting a failure in informed consent. Even if the surrogates have consented to surrogacy, it does not follow that they have therefore consented to *any such following procedure*. Following Onora O’Neill, consent is not transitive: “I may consent to A, and A may entail B, but if I am blind to the entailment I need not consent to B.”^87^ Thus, while surrogates may ostensibly have consented to surrogacy, A, this does not entail that they have consented to sub-sequent practices, B, such as foetal reduction and a caesarean delivery, which appear to be entailed by surrogacy in the Indian context.

However, that surrogates give proper informed consent to A, let alone any entailments of A, is unclear. Pande found that the surrogacy contract is in English, a language almost none of the surrogates can read.^88^ Some essential points of the contract are translated for them, and one surrogate states that, “the only thing they told me was that this thing is not immoral, I will not have to sleep with anyone, and that the seed will be transferred into me with an injection,” whilst another adds, “we were told that if anything happens to the child, it’s not our responsibility but if anything happens to me, we can’t hold anyone responsible.”^89^ Another surrogate, interviewed in Anand, Gujurat, says: “We are not really told much about the medicines and injections… We [her husband and she] are not as educated as you are, you know. I won’t really understand much else! And I trust Doctor Madam, so I don’t ask.”^90^ Evidence suggests that the surrogacy procedure has been oversimplified, to surrogates who are uneducated and do not seem to understand what the medical procedures entail. There appears to be no mention of the various risks associated with pregnancy and surrogacy, and one surrogate’s comments indicate that the contract involves the surrogate waiving the clinic’s responsibility for her wellbeing if something were to go wrong. It is hard to see how this could be validly consented to if the risks associated with the procedures have not been appropriately explained or understood.

Informed consent is central to discussions on ethically acceptable medical practice. It is seen as necessary ethical justification for medical treatment, research on human subjects, and uses of human tissues.^91^ While some associate it with respecting a patient’s autonomy, O’Neill points out that its importance is more elementary: it provides reasonable assurance that a patient has not been deceived or coerced.^92^ O’Neill acknowledges that “genuine consent is not a matter of overwhelming patients with information, arrays of boxes to tick or propositions for signature.”^93^ Rather, it is apparent where “patients can *control* the amount of information they receive, and what they allow to be done.”^94^ Giving patients control in this way could consist in “offering easy access to more specific information that lies behind an initial, or second, or third layer of information provided.”^95^ The key idea is that additional accurate information is readily available as demanded.

While surrogates have not been presented with a wealth of information, this need not be indicative of a lack of consent: following O’Neill, an overabundance of information is not necessary for consent. Could, therefore, the above narrative describe a situation in which surrogates have been able to *control* the amount of information they receive, and what they allow to be done? It appears not. There is also a general level of deference to the medical profession that is particularly heightened in the context of Indian surrogacy, where the women in question lack education and basic reproductive knowledge. It seems implausible that such women would ask for further information, or that medical professionals would be willing to provide it on demand, as earlier evidence suggests that crucial details relating to surrogacy have been withheld and oversimplified. These problems are primed to resurface in the context of UTx. In the case of uterine sales, the pool of vendors is likely to emulate the pool of women who have typically acted as gestational surrogates: those in socioeconomic need, from a disadvantaged cross-section of society, similarly lacking in education. Issues such as an inability to understand the contract, control the amount of information received, a lack of medical knowledge, and deference displayed to the medical profession are therefore likely to persist.

### 4.2 Unique nature of uterine donation

In the case of uterine *donation*, as opposed to sale, similar failures in consent could arise as a result of the unique nature of UTx. Given that UTx remains a highly experimental procedure, with the risks and benefits currently defying standard or conclusive calculation, the information provided to donors and recipients in the Indian context is unlikely to meet standards of informed consent. This is because the nature of the doctor-patient relationship in India appears to be one in which clinicians control the information that is disclosed, and patients do not typically feel able to ask for more, as has been observed in the surrogacy context. The experimental nature of UTx also means that its associated risks and harms are perhaps harder to calculate; thus, patients are in an even weaker position in terms of making this calculation for themselves and for giving informed consent.

Although UTx also poses unique risks and harms that need to be understood by the organ *recipient* (who may also face information failures in the Indian context), I focus on the risks and harms to the organ *donor*.^96^ Risks specific to uterine donation include the physical and psychological harms associated with the permanent loss of the uterus. While livers can be regenerated and a single kidney can be donated without much clinical significance, a uterus cannot be regenerated. Some of the reported consequences following hysterectomy include a loss of gender identity, negative effects on libido and sexual satisfaction, and an increase in sexual dysfunction.^97^ Issues of gender identity and sexual satisfaction are closely tethered to an individual’s everyday experience, relationships, and sense of self, and thus plausibly have a real bearing on a donor’s quality of life after the transplant.^98^ The authors of the Montreal Criteria argue that “to ensure that prospective donors make informed, autonomous decisions, there is an *added impetus* to give a potential uterus donor both *comprehensive information* relating to giving up a healthy uterus and time to consider such a *significant and irrevocable decision*.”^99^

There is reason to believe that the disclosure of *comprehensive information* may be hard to ensure in the Indian context. Firstly, it has been observed in the surrogacy context that the individuals who might be likely to enter into such contracts have limited medical understanding and may not be able to fully comprehend the risks associated with UTx donation. Further, clinicians’ perceptions that such understanding is beyond the donors’ capabilities might mean that they routinely do not offer such information, believing such disclosure to be futile. Moreover, the general deference displayed towards the medical profession means that even if the risks *were* outlined, donors might absolve themselves of any decision-making capacity, leaving such decisions in the hands of clinicians.

### 4.3 Hysterectomy use in India

Evidence on the routine use of medically unnecessary hysterectomies in India provides further reason to believe that the risks associated with uterine donation are not likely to be sufficiently disclosed to donors in the case of UTx. Patient autonomy and informed consent do not seem to be primary concerns of many clinicians operating in this context. There is significant evidence to suggest that doctors are performing unnecessary hysterectomies on impoverished rural women for commercial gain.^100^ Investigative reporting found that women are “frightened” into having hysterectomies by private doctors who use these operations as opportunities to tap into funds from the government’s national health insurance scheme. Implemented to assist those living below the poverty line, the scheme allows families to receive treatments up to a certain cost, from designated private hospitals, which then claim the costs directly from the state. Invasive procedures, such as surgeries, earn the private hospitals more than less invasive treatments, thus generating an incentive to perform unnecessary surgical procedures. One reporter found that when she enquired as to how many women in a particular village had undergone hysterectomies, “more than half raised their hands at once. Village leaders said about 90% of the village women have had the operation, including many in their 20s and 30s.”^101^

A 2016 study published in the Indian Journal of Medical Ethics found:

doctors provided grossly unscientific information to poor Dalit women to instil a fear of “cancer” in their minds to wilfully mislead them to undergo hysterectomies, following which many suffered complications and died… a large proportion of the hysterectomies performed were medically unwarranted; that private doctors were using highly suspect diagnostic criteria, based on a single ultrasound scan, to perform the hysterectomies and had not sent even a single sample for histopathology; and that the medical records were incomplete, erroneous and, in several instances, manipulated.^102^

The researchers suggest that this situation is a result of a combination of “patriarchal bias, professional unscrupulousness and pro-private healthcare policies.”^103^ A 2018 survey found that about 3 per cent of all Indian women had undergone a hysterectomy, mostly uneducated, rural women, including teenagers.^104^

One woman describes the incident the following way:

I went to the clinic because I had heavy bleeding during menstruation,” she says.

The doctor did an ultrasound and said I might develop cancer. He rushed me into having a hysterectomy that same day. Sunita says she was reluctant to have the operation straightaway and wanted to discuss it with her husband first. She says the doctor said the operation was urgent and sent her for surgery just hours later.^105^

These procedures are apparently performed without the patient sufficiently understanding the nature of the procedure or the reasons it might be required, let alone after their having received sufficient guidance on the potential psychosexual repercussions. There is a tendency for doctors operating in this context to display an unscrupulousness that might undermine the goal set by the Montreal criteria of providing *comprehensive information*.

These findings align with the earlier observation on coerced sterilisations and the fact that it is a certain class of women being consistently subjected to invasive and unnecessary medical interventions. There could plausibly be a ready supply of uteri sourced from women who are typically targeted for sterilisation programmes and unnecessary hysterectomies. The potential for the merging of these three goals – uterine procurement for UTx, private hospital enrichment, and population control – should motivate concern. Regulators ought to be mindful of the targeting of this population for sterilisation and hysterectomies when considering potential consequences of the widespread development of UTx, and consider which women might be most likely to serve as donors.

Accordingly, the consent requirement set out by the Montreal criteria, requiring comprehensive information and that the donor “is responsible enough to consent, informed enough to make a responsible decision, and not under coercion,”^106^ might be difficult to satisfy in the Indian context, due to the precedents outlined for flouting such standards. Importantly, informed consent would be lacking even under an altruistic uterine procurement model. This is because key factors in determining the level of information disclosed to patients seems to be the deference shown to the medical profession and a lack of general understanding about medical procedures. Such factors are likely to remain in the context of altruistic UTx and thus lead to attendant information failures. It is also important to note that uterus donation is reportedly “much more complicated than even a radical hysterectomy because long veins and arteries must be removed.”^107^ As a result, the attendant risks and harms of the procedure are heightened. This increased risk, and indeed difficulty in explaining the risk, may further obstruct the goal of obtaining proper informed consent.

## 5 Conclusion

Given recent global trends in assisted reproductive technologies and transplant tourism, uterine donation may also grow to be regulated by market norms. Drawing a parallel with commercial surrogacy, and the way in which it has operated in a typically exploitative manner, I have suggested that uterine sales would display similar features of exploitation. This is because despite the outlawing of commercial surrogacy, certain key structural conditions remain in place, relating to the vulnerability of surrogates and the profit-driven and deregulated nature of the Indian ART context. As a result, we ought to be mindful of the potential for exploitation regarding uterine sales. Further, these structural factors intersect with factors peculiar to UTx, such as its experimental nature, as well as the novel risks and harms it poses, and thus contribute to the difficulty in obtaining proper informed consent. Importantly, such failures in consent arise in the context of altruistic donation too. It has been my aim to pay due regard to the empirical reality surrounding the Indian healthcare context in order to provide a context-sensitive ethical analysis of UTx that attends to how it is primed to develop in this setting. I have concluded that, in light of these systemic concerns, UTx in India is unlikely to meet the Montreal criteria for the ethical feasibility of uterine transplantation.

## Footnotes

1 Knapton, S. (2019, April 9). Baby makes history after world’s first robotic womb transplant. *The Telegraph*. Retrieved from https://www.telegraph.co.uk/science/2019/04/09/first-baby-born-donated-womb-grandmother-little-help-robot/.

2 Wilkinson, S., & Williams, N.J. (2016). Should uterus transplants be publicly funded? *Journal of Medical Ethics*. *42*(9), 559–565. https://doi.org/10.1136/medethics-2015-102999.

3 Ibid.

4 Srinivasan, S. (2017). Uterus transplants in India: yawning regulatory gaps. *Indian Journal of Medical Ethics*. https://doi.org/10.20529/IJME.2017.066.

5 Where such exploitation constitutes a harm, on the distinction put forward by Alan Wertheimer. Wertheimer, A. (1992). Two questions about surrogacy and exploitation. *Philosophy & Public Affairs*. 211–239.

6 Teena, X., Akhila, V., & Vijayakumar, S. (2017). Instilling fear makes good business sense: unwarranted hysterectomies in Karnataka. *Indian Journal of Medical Ethics*. *2*(1), 49–55.

7 Lefkowitz, A., Edwards, M., & Balayla, J. (2012). The Montreal Criteria for the Ethical Feasibility of Uterine Transplantation. *Transplant International: Official Journal of the European Society for Organ Transplantation*. *25*(4), 439–447. https://doi.org/10.1111/j.1432-2277.2012.01438.x.

8 Ibid., p. 440.

9 Ibid., p. 442.

10 Gallagher, J. (2018, December 5). First baby born after deceased womb transplant. Retrieved from https://www.bbc.com/news/health-46438396

11 Brännström, M., Dahm Kähler, P., Greite, R., Mölne, J., Díaz-García, C., & Tullius, S.G. (2018). Uterus Transplantation: A Rapidly Expanding Field. *Transplantation*. *102*(4), 569–577. https://doi.org/10.1097/TP.0000000000002035.

12 Williams, N. (2016). Should Deceased Donation be Morally Preferred in Uterine Transplantation Trials? *Bioethics*. *30*(6), 415–424. https://doi.org/10.1111/bioe.12247.

13 Ibid., p. 417.

14 Ibid.

15 ”Since most potential kidney vendors will never become unpaid donors, either during life or posthumously, the prohibition of sales must be presumed to exclude kidneys that would otherwise be available. It is therefore essential to make sure that there is adequate justification for the resulting harm.” Radcliffe-Richards, J., Daar, A., Guttmann, R., et al. (1998). The case for allowing kidney sales. *The Lancet*. *351*(9120), 1950–1952. https://doi.org/10.1016/S0140-6736(97)08211-1.

16 Erin, C.A. (2003). An ethical market in human organs. *Journal of Medical Ethics. 29*(3), 137–138. https://doi.org/10.1136/jme.29.3.137.

17 Ghods, A.J., & Savaj, S. (2006). Iranian Model of Paid and Regulated Living-Unrelated Kidney Donation. *Clinical Journal of the American Society of Nephrology*. *1*(6), 1136–1145. https://doi.org/10.2215/CJN.00700206.

18 Arvidsson, A., Johnsdotter, S., & Essén, B. (2015). Views of Swedish Commissioning Parents Relating to the Exploitation Discourse in Using Transnational Surrogacy. *PLOS ONE*. *10*(5), e0126518. https://doi.org/10.1371/journal.pone.0126518.

19 Brännström, Dahm Kähler, Greite, Mölne, Díaz-García, Tullius, op. cit. note 11, pp. 569–577.

20 H, G. (n.d.). First successful uterus transplant in the MENA region was completed in Lebanon! Retrieved May 17, 2019, from https://www.the961.com/news/uterus-transplant-in-lebanon.

21 Surrogacy not an option in Lebanon | News, Lebanon News | THE DAILY STAR. (n.d.). Retrieved May 17, 2019, from http://www.dailystar.com.lb/News/Lebanon-News/2014/Sep-13/270527-surrogacy-not-an-option-in-lebanon.ashx.

22 Egg donation is now legal but may face challenges | News, Lebanon News | THE DAILY STAR. (n.d.). Retrieved May 17, 2019, from http://www.dailystar.com.lb/News/Lebanon-News/2004/Mar-16/348-egg-donation-is-now-legal-but-may-face-challenges.ashx

23 Khalife, J., Rafeh, N., Makouk, J., et al. (2017). Hospital Contracting Reforms: The Lebanese Ministry of Public Health Experience. *Health Systems & Reform*. *3*(1), 34–41. https://doi.org/10.1080/23288604.2016.1272979.

24 Forsyth, A. (2017, April 25). The organ trafficker who preys on refugees. Retrieved from https://www.bbc.com/news/magazine-39272511.

25 Hans. (2018, January 22). Lebanon [Text]. Retrieved May 17, 2019, from https://ec.europa.eu/echo/where/middle-east/lebanon_en.

26 India’s first uterine transplant baby born: What is a womb transplant? | Health Tips and News. (n.d.). Retrieved May 20, 2019, from https://www.timesnownews.com/health/article/indias-first-uterine-transplant-baby-born-what-is-a-womb-transplant/301697.

27 Srinivasan, op. cit. note 4, p. 136.

28 Ibid.

29 Ministry of Health and Family Welfare. (2014). Transplantations of Human Organs and Tissues Rules. Retrieved June 19, 2019, from https://notto.gov.in/act-end-rules-of-thoa.htm.

30 Vora, P. (n.d.). Uterus transplants are experimental and risky but Indian women are already lining up for them [Text]. Retrieved June 5, 2020, from https://scroll.in/pulse/837833/uterus-transplants-are-experimental-and-risky-but-indian-women-are-already-lining-up-for-them.

31 Srinivasan, op. cit. note 4, p. 137.

32 Ibid.

33 Wilkinson, S. (2016). Exploitation in International Paid Surrogacy Arrangements. *Journal of Applied Philosophy*. *33*(2), 125–145. https://doi.org/10.1111/japp.12138.

34 Rudrappa, S. (n.d.). India outlawed commercial surrogacy – clinics are finding loopholes. Retrieved June 5, 2019, from http://theconversation.com/india-outlawed-commercial-surrogacy-clinics-are-finding-loopholes-81784.

35 WHO | The state of the international organ trade: a provisional picture based on integration of available information. (n.d.). Retrieved June 5, 2019, from https://www.who.int/bulletin/volumes/85/12/06-039370/en/.

36 Chugh, N. (2015). Need a kidney? Inside the world’s biggest organ market. *Al Jazeera*.Retrieved from https://www.aljazeera.com/indepth/features/2015/10/kidney-worlds-biggest-organ-market-151007074725022.html.

37 India bans commercial surrogacy to stop ‘rent a womb’ exploitation of vulnerable women. (n.d.). Retrieved May 20, 2019, from https://www.telegraph.co.uk/news/2018/12/20/india-bans-commercial-surrogacy-stop-rent-womb-exploitation/.

38 Bindel, J. (2016, April 1). Outsourcing pregnancy: a visit to India’s surrogacy clinics | Julie Bindel. *The Guardian*. Retrieved from http://www.theguardian.com/global-development/2016/apr/01/outsourcing-pregnancy-india-surrogacy-clinics-julie-bindel.

39 Surrogacy Fees & Costs. (n.d.). Retrieved January 3, 2018, from https://www.surrogacyamerica.com/prospective-parents/surrogacy-cost-fees.

40 Surrogacy Arrangements Act 1985. (n.d.). [Text]. Retrieved June 16, 2016, from http://www.legislation.gov.uk/ukpga/1985/49.

41 No commercial surrogacy, only for needy Indian couples, Govt tells SC. (2015, October 29). *The Indian Express*. Retrieved from http://indianexpress.com/article/india/india-news-india/govt-to-make-commercial-surrogacy-illegal-panel-to-decide-on-cases-of-infertile-couples/.

42 Reuters. (2014). India Slashes Health Budget, Already Among World’s Lowest: Report. *NDTV.Com*. Retrieved from http://www.ndtv.com/india-news/india-slashes-health-budget-already-among-worlds-lowest-report-717235

43 Health expenditure, total (% of GDP) | Data | Table. (n.d.). Retrieved June 16, 2016, from http://data.worldbank.org/indicator/SH.XPD.TOTL.ZS.

44 Unnithan, M. (2011). Infertility and Assisted Reproductive Technologies (ARTs) in a Globalising India: Ethics, Medicalisation and Agency. *Asian Bioethics Review*. *2*(1), 3–18.

45 PRS | Bill Track | The Surrogacy (Regulation) Bill, 2016. Retrieved from http://www.prsindia.org/billtrack/the-surrogacy-regulation-bill-2016-4470/.

46 Gallie, W.B. (1955). Essentially Contested Concepts. *Proceedings of the Aristotelian Society*. *56*, 167–198.

47 Zwolinski, M., & Wertheimer, A. (2016). Exploitation. In E.N. Zalta (Ed.), The Stanford Encyclopedia of Philosophy (Fall 2016). Metaphysics Research Lab, Stanford University. Retrieved from https://plato.stanford.edu/archives/fall2016/entries/exploitation/.

48 Ibid.

49 Ibid.

50 This would exclude cases such as a ‘vulnerable child,’ where the parent uses this vulnerability to instil discipline in the child. The child is not vulnerable in the sense relevant to justice, and therefore to exploitation, on this account.

51 Wertheimer, op. cit. note 5, pp. 211–239.

52 Pande, A. (2010). Commercial Surrogacy in India: Manufacturing a Perfect Mother-Worker. *Signs*. *35*(4), 969–992. https://doi.org/10.1086/651043, p. 974.

53 Pande, op. cit. note 52, pp. 969-992.

54 Pande, Amrita. (2014). Wombs In Labour: Transnational Commercial Surrogacy in India. New York: Columbia University Press, p. 20.

55 Ibid.

56 Ibid.

57 Deomampo, D. (2013). Transnational Surrogacy in India: Interrogating Power and Women’s Agency. *Frontiers: A Journal of Women Studies*. *34*(3), 167–188. https://doi.org/10.5250/fronjwomestud.34.3.0167.

58 Reddy, S., & Patel, T. (2015). “There are many eggs in my body”: medical markets and commodified bodies in India. *Global Bioethics*. *26*(3–4), 218–231. https://doi.org/10.1080/11287462.2015.1112625, p. 221.

59 Ibid.

60 Reddy & Patel, op. cit. note 58, pp. 218–231.

61 Rudrappa, S. (2015). Discounted Life: The Price of Global Surrogacy in India. New York: New York University Press, p. 24.

62 Pande & Amrita, op. cit. note 58, p. 112.

63 Rudrappa, op. cit. note 61, p. 26.

64 Ibid.

65 Ledbetter, R. (1984). Thirty Years of Family Planning in India. *Asian Survey. 24*(7), 736–758. https://doi.org/10.2307/2644186.

66 Hartmann, B. (1995). Reproductive Rights and Wrongs: The Global Politics of Population Control. Cambridge, MA: South End Press.

67 Narayana, G., & Kantner, J.F. (1992). Doing the Needful: The Dilemma of India’s Population Policy. Boulder, CO: Westview., p. 111.

68 This approach to population control does not appear to have been relegated to the past. In 2012, the UK gave India £166m to fund a sterilisation programme, which predominantly targeted the poor. It was reported that people were threatened with losing their ration cards if they did not undergo sterilisation, or were incentivised to participate by being given 600 rupees (around £7) and a sari. Some states reportedly also ran lotteries in which people could win cars and fridges. Indeed, it was reported in October 2016 that such state-run sterilisation camps still exist. Chamberlain, G. (2012, April 15). UK aid helps to fund forced sterilisation of India’s poor. *The Guardian*. Retrieved from https://www.theguardian.com/world/2012/apr/15/uk-aid-forced-sterilisation-india.

69 Scheper-Hughes, N. (2003). Keeping an eye on the global traffic in human organs. *The Lancet*. *361*(9369), 1645–1648. https://doi.org/10.1016/S0140-6736(03)13305-3.

70 ART Bill may close surrogacy doors for foreigners, unmarried people - The Hindu. (n.d.). Retrieved June 7, 2016, from http://www.thehindu.com/news/cities/Delhi/art-bill-may-close-surrogacy-doors-for-foreigners-unmarried-people/article7793884.ece.

71 Wertheimer, op. cit. note 5, p. 216.

72 Multiple pregnancy: antenatal care for twin and triplet pregnancies | Guidance and guidelines | NICE. (n.d.). Retrieved October 28, 2017, from https://www.nice.org.uk/guidance/cg129/chapter/introduction.

73 Criteria for number of embryos to transfer: a committee opinion. (2013). *Fertility and Sterility*. *99*(1), 44–46. https://doi.org/10.1016/j.fertnstert.2012.09.038.

74 Ahmad, T. (2015, November 2). India: Draft Legislation Regulating Assisted Reproductive Technology Published |Global Legal Monitor [web page]. Retrieved October 28,2017, from //www.loc.gov/law/foreign-news/article/india-draft-legislatio n-regulating-assisted-reproductive-technology-published/.

75 Carney, S. (2011) The Red Market. New York: Harper Collins, p. 148.

76 Multifetal Pregnancy Reduction-Topic Overview. (n.d.). Retrieved October 28, 2017, from https://www.webmd.com/infertility-and-reproduction/tc/multifetal-pregnancy-reduction-topic-overview.

77 Carney, op. cit. note 75.

78 Ibid.

79 India surrogacy: Chapter Three - San Francisco Chronicle. (n.d.). Retrieved October 28, 2017, from http://www.sfchronicle.com/local/bayarea/item/India-surrogacy-3-24058.php.

80 Pande & Amrita, op. cit. note 58, p. 117.

81 Rudrappa, op. cit. note 67, p. 115.

82 Sahay, M. (2018). Transplantation of human organs and tissues Act-”Simplified”. *Indian Journal of Transplantation*. *12*(2), 84. https://doi.org/10.4103/ijot.ijot_31_18.

83 India’s 1st womb transplant ‘dangerous escapade of surgical cowboys’, warns doc. (2017, May 18). Retrieved June 5, 2019, from https://www.hindustantimes.com/health/dangerous-escapade-of-surgical-cowboys-pioneer-doctor-on-india-s-1st-uterus-transplant/story-5fpAQnpxMOvtRoMArOYL6H.html.

84 Srinivasan, op. cit. note 4, p. 186.

85 Teena, Akhila & Vijayakumar, op. cit. note 6, pp. 49–55.

86 Lefkowitz, Edwards & Balayla, op. cit. note 7, pp. 439–447.

87 O’Neill, O. (2003). Some limits of informed consent. *Journal of Medical Ethics. 29*(1), 4–7. https://doi.org/10.1136/jme.29.1.4

88 Pande, op. cit. note 55, p. 976.

89 Ibid.

90 Pande, A. (2009). Not an ‘Angel’, not a ‘Whore’: Surrogates as ‘Dirty’ Workers in India. *Indian Journal of Gender Studies*. *16*(2), 141-173. https://doi.org/10.1177/097152150901600201, p. 148.

91 O’Neill, op. cit. note 87, pp. 4–7.

92 Ibid., p. 5.

93 Ibid., p. 6.

94 Ibid.

95 Ibid.

96 However, it may well be the case that the risks and harms related to the recipient (such as the low chances of carrying and giving birth to a healthy baby, the fact that the pregnancy will not be an *ordinary* pregnancy and that foetal movements and contractions will not be felt, and caesareans sections will be required) have a bearing on the cost-benefit calculation the donor makes. For example, a donor might decide it is not worth putting themselves through the necessary harms of donation once they realise that the recipient would have such low chances of pregnancy. Nevertheless, I shall set this possibility aside and focus only on donor-specific harms.

97 Ferroni, P., & Deeble, J. (1996). Women’s subjective experience of hysterectomy. *Australian Health Review: A Publication of the Australian Hospital Association*. *19*(2), 40–55.

98 It might be interjected that the aforementioned identity and sexual dysfunction harms might be more likely to occur when a hysterectomy is undesired, medically indicated, or forced. In the case of perceived-voluntary uterus donation, however, if consent conditions are met, these harms might appear far less pronounced, compared with the harms experienced by women who did not want to lose their uteri, but were required to as treatment. There is presently insufficient evidence comparing the level of harm experienced by these groups, so it is not clear whether psychosexual harms are themselves dependent on the way in which the hysterectomy is viewed by the patient. It may be the case that such harms result from the procedure itself and the resultant bodily changes.

99 Lefkowitz, Edwards & Balayla, op. cit. note 7, p. 443, [emphasis added].

100 McGivering, J. (2013, February 6). India’s hysterectomy scam. Retrieved from https://www.bbc.com/news/magazine-21297606.

101 Ibid.

102 Teena, Akhila & Vijayakumar, op. cit. note 6, pp. 49–55.

103 Ibid.

104 Private doctors perform most hysterectomies in India: survey. (2018, January 11). *Reuters*. Retrieved from https://www.reuters.com/article/us-india-women-health-idUSKBN1F02GJ

105 McGivering, op. cit. note 102.

106 Lefkowitz, Edwards & Balayla, op. cit. note 7, p. 444.

107 Robertson, J.A. (2016). Other women’s wombs: uterus transplants and gestational surrogacy. *Journal of Law and the Biosciences*. *3*(1), 68–86. https://doi.org/10.1093/jlb/lsw011, p. 71.

